# The modified Blumgart anastomosis after pancreaticoduodenectomy: a retrospective single center cohort study

**DOI:** 10.1515/iss-2020-0021

**Published:** 2020-12-21

**Authors:** Georgi Kalev, Christoph Marquardt, Herbert Matzke, Paul Matovu, Thomas Schiedeck

**Affiliations:** Department of General, Visceral, Thoracic and Pediatric Surgery, Ludwigsburg Hospital, Ludwigsburg, Germany

**Keywords:** Blumgart technique, pancreatic anastomosis, pancreatic fistula, pancreaticojejunostomy, propensity score matching, resection of the pancreatic head

## Abstract

**Objectives:**

The postoperative pancreatic fistula (POPF) is a major complication after pancreatic head resection whereby the technique of the anastomosis is a very influencing factor. The literature describes a possible protective role of the Blumgart anastomosis.

**Methods:**

Patients after pancreatic head resection with reconstruction through the modified Blumgart anastomosis (a 2 row pancreatic anastomosis through mattress sutures of the parenchyma and duct to mucosa pancreaticojejunostomy, Blumgart-group) were compared with patients after pancreatic head resection and reconstruction through the conventional pancreatojejunostomy (single suture technique of capsule and parenchyma to seromuscularis, PJ-group). The Data were collected retrospectively. Depending on the propensity score matching in a ratio of 1:2 comparison groups were set up. Blumgart-group (n=29) and PJ-group (n=56). The primary end point was the rate of POPF. Secondary goals were duration of operation, length of hospital stay, length of stay on intermediate care units and hospital mortality.

**Results:**

The rate of POPF (biochemical leak, POPF “grade B” and POPF “grade C”) was less in the Blumgart-group, but without statistical relevance (p=0.23). Significantly less was the rate of POPF “grade C” in the Blumgart-group (p=0.03). Regarding the duration of hospital stay, length of stay on intermediate care units and hospital mortality, there was no relevant statistical difference between the groups (p=0.1; p=0.4; p=0.7). The duration of the operation was significantly less in the Blumgart-group (p=0.001).

**Conclusions:**

The modified Blumgart anastomosis technique may have the potential to decrease major postoperative pancreatic fistula.

## Introduction

The pancreatic head resection is the only form of therapy with curative intention for patients with pancreas carcinoma. Diverse cystic pancreatic lesions could also define surgical indication depending on their malignant potential. The latter have been more and more coincidentally diagnosed because of the increased use of imaging; in the end, this has led to an increase in the number of operations and hence an increased relevance of the postoperative complications. Anastomotic leakage (POPF) belongs to the major postoperative complications and has a big impact on the postoperative outcome. The technique of the anastomosis plays an important role in the appearance of localized inflammation and hence occurrence of POPF. The goal of this study is to evaluate the difference in occurrence of POPF after Blumgart anastomosis in comparison to the conventional pancreatojejunostomy in our center. Furthermore, the duration of the operation, the hospital mortality and length of stay on the intermediate care units will be assessed. Propensity score matching (PSM) will be used to counter the disadvantage of retrospective data analysis.

## Materials and methods

### Patients

Between 01/2010 and 02/2020, 221 Patients underwent a pancreatic head resection. The modified Blumgart anastomosis was carried out in 32 of the patients. Patients with malignant and benign lesions of the pancreatic head were included in the study. Emergency operations and duodenum-preserving pancreatic head resections (Berger’s and Frey’s procedure) were excluded. After propensity score matching in a ratio of 1:2 29 Patients remained in the Blumgart group and 56 patients in the control group. The data were collected retrospectively from patient files in our digital hospital archive.

We are a certified pancreatic center of the German Cancer Society (DGK). As we are a referral hospital in a holding of 9 hospitals (RKH Regional Hospital Holding) in the south of Germany, all pancreatic resections of this area are referred to our pancreas center, and we performed on average 34 pancreatic resections (24 resections for cancer) per year since 2017. Thus with our case load, the mortality rate was always lying below the required 5% for certification. Only 2 pancreatic surgeons are certified and are present during every pancreatic resection.

### Technique of the anastomosis

The pancreatic anastomoses are inconsistently defined in the literature. Although our reference anastomosis of the pancreatic parenchyma with the jejunum (pancreatojejunostomy) is often combined with single sutures of the pancreatic duct (pancreaticojejunostomy), in the following description we will be using the term pancreatojejunostomy. The locational descriptions are defined according to the anatomical descriptions in relation to the whole body. First of all, the dissected jejunum-loop will be pulled up retrocolic without tension through the transverse mesocolon.

Until September 2017 the conventional end-to-side pancreatojejunostomy was the standard in our center (PJ-group), we suture the anterior-, and posterior wall in interrupted suture technique, thus the capsule and parenchyma are fixed to the seromuscularis of the jejunum using 3-0 braided Vicryl^™^ (Ethicon, Inc.) sutures. Since the pancreatic duct lies eccentrically dorsal, its dorsal wall will also be fixed with one, rarely with 2–3 of the stitches through the parenchyma to form the pancreaticojejunostomy. Next the anterior wall is sutured in the same interrupted suture technique. If needed, intermediate stitches can now be brought in. Our anastomosis corresponds to the pancreaticojejunostomy described by Bassi 2003 [1], [2].

For the modified Blumgart anastomosis (Blumgart-group) [3], we place two transpancreatic mattress sutures. For this two double-armed monofilament PDS 3-0 sutures are used. Subsequently, we perform the pancreaticojejunostomy with 4 sutures (PDS 5-0). From September 2017 until today, the modified Blumgart anastomosis represents the technical standard of performing the anastomosis in our hospital.

### Study endpoints and definitions

The primary endpoint of the study is the rate of postoperative pancreatic fistula (POPF). During the retrospective data research in this work, postoperative fistula were classified as followed according to the latest definitions [4]. The International study Group on Pancreatic Surgery (ISGPS) 2016 defines POPF as an abnormal communication between the pancreatic duct system and another epithelial surface, containing pancreas-derived, enzyme-rich fluid, in addition on or after postoperative day 3 with amylase level >3 times the upper limit of normal amylase from the drainage system. There are 3 severity levels of the POPF. Biochemical leak (earlier grade A in the definition from 2005 [5]) refers to a drain output of any measurable volume of fluid on or after postoperative day 3 with an amylase content greater than 3 times the serum amylase activity, which can persist up to 3 weeks after the operation. A biochemical leak has no clinical relevance and is therefore strictly speaking not a POPF. Actually recognized as POPF are grade B and grade C, where grade B POPF is characterized by a change in the postoperative management. A drain for more than 3 weeks or interventional/endoscopic placement of a drainage system may be necessary. grade C POPF leads to organ failure, reoperation and/or death [4].

The duration of the operation, the length of stay on general wards, the length of stay on intermediate care units and the hospital mortality will be assessed as secondary endpoints.

### Statistical analysis

By normally distributed data we used the mean and the standard deviation for the presentation of the central tendency and the measure of variation, by the non-normally distributed data we used accordingly the median as well as the 1st and third quartiles. The testing of data on normal distribution was done with the Kolmogorov-Smirnov-Test or with the Shapiro–Walk–Test. The Mann–Whitney–U Test was used to prove the zero hypotheses on non-normally distributed Data. The chi-square test was used for the nominal data and the Fisher’s exact test was used for expected frequencies below 5. We chose p<0.05 as the level of significance.

By Thoemmes developed R-code and custom dialog for SPSS were used for the PSM [6]. His program psmatching used the following R-Packets – “Matching” [7], “RItools” [8] und “cem” [9].

## Results

### Propensity score matching (PSM). Clinical and demographical patient factors after pairing

ASA score, BMI and age were used as covariables for the PSM. Intraoperative blood transfusion was taken as a sign of massive intraoperative blood loss. Other confounders which are known to be risk factors for a POPF are: Pancreatic duct of small caliber (<3 mm) and soft pancreatic tissue [5]. These factors were not clearly documented in the available patient files. Therefore, their retrospective evaluation was impossible. A matching algorithm of “next neighbor” with a ratio of Blumgart-group : PJ-group=1:2 was chosen. A caliper from 0.2 of the standard deviations of the logit of the propensity score was also chosen.

After matching 29 patients from the Blumgart-group and 56 Patients from the PJ-group remained for analysis (for 3 Patients from the Blumgart-group there were no corresponding Patients from the control group found and for 2 Patients from the Blumgart-group there was only one adequate neighbor from the control group).

The clinical and demographical patient factors (Table 1) show a homogenous distribution in the paired groups. Intraoperative blood transfusion is taken as a sign of massive blood loss. After the operation malignancy was histologically confirmed in 38 patients of the PJ-group and 21 Patients from the Blumgart-group. The diagnosis of carcinoma was not taken as inclusion or exclusion criteria.

**Table 1:** Clinical and demographical factors of the paired patients.

	Age	Sex f/m	ASA score				BMI	Blood transfusion	Pancreatitis
			I	II	III	IV			
PJ-group (control group) (n=56)	69^a^ (57/76)^b^	22/34	0	28	28	0	25^a^ (23/29)^b^	8^c^	7^c^
Blumgart-group (n=29)	64^a^ (57/78)^b^	11/18	0	13	16	0	27^a^ (22/29)^b^	4^c^	5^c^

^a^Median. ^b^First Quartile/third Quartile. ^c^Patients.

### Study endpoints

All study endpoints are shown in Table 2

**Table 2:** Study endpoints. Comparison of the groups and significance of the difference.

Study endpoint	PJ-group	Blumgart-group	Significance
Total no. of patients	56	29	
Total-POPF	14^a^	4^a^	p=0.23
POPF-“Grad C”	12^a^	1^a^	p=0.03
Duration of the OP	310.5 min (270/366 min)^b^	266 min (240/297 min)^b^	p=0.001
Hospital stay	22 days (15/28 days)^b^	16 days (14/23 days)	p=0.1
ICU stay	2 days (1/7 days)^b^	2 days (2/7 days)^b^	p=0.4
Postoperative death	6^a,c^	4^a,c^	p=0.7

^a^Patients. ^b^Median (1/3 Quartile). ^c^As we did not analyze statistically representative samples but only matched subgroups with certain demographic and clinical characteristics, the calculation of a mortality rate in this context does not correspond to the total mortality rate, which in our pancreatic center lies below the required 5% for certification. We reviewed every single patient chart of our subgroups thoroughly and we found no evidence for any “failure to rescue”.

The rate of POPF was selected as the primary endpoint. On 14 Patients in the PJ-group a POPF (“biochemical Leak”, POPF “grade B” and POPF “grade C”) was discovered, meanwhile 4 patients in the Blumgart-group developed a POPF. The chi-square test showed no significant difference (p=0.23). By the occurrence of the clinically most significant POPF grade C, the exact test after Fisher showed a clinically significant difference (12 in the PJ-group vs. 1 in the Blumgart-group) (p=0.03).

The median duration of the operation in the PJ-group was 310.5 min (1/3 Quartile-270/366 min). In the Blumgart-group this was 266 min (1/3 Quartile-240/297 min). The Mann–Whitney–U Test showed a significant statistical difference (p=0.001).

The median hospital stay was 22 days in the PJ group (1/3 Quartile-15/28 days) vs. 16 days (1/3 Quartile 14/23 days) in the Blumgart-group. The Mann–Whitney–U Test showed no significant statistical difference (p=0.1).

The Patients from the PJ-group stayed 2 days in average on the intermediate care units (1/3 Quartile-1/7 days). In the Blumgart-group the median stay was also 2 days (1/3 Quartile-2/7 days). Deceased Patients were excluded. There was no significant difference between the 2 groups (Mann–Whitney–Test; p=0.4).

Six Patients in the control group and 4 patients in the Blumgart group died from a surgical or non-surgical cause. The chi-square test showed no statistically significant difference (p=0.7).

## Discussion

The incidence of POPF after pancreas resection is estimated in a range between 9.9% and 28.5% depending on the definition [10], [11], [12]. By definition it is one of the most important factors responsible for postoperative morbidity and mortality. It is often associated with abscess formation eventually with abdominal sepsis or life threatening erosional bleeding. The clinically relevant POPF lead secondarily to prolonged hospital stay and longer stay on intermediate care wards, which influences the total treatment costs [13]. The risk factors include soft pancreatic parenchyma, pancreatic lesions without pancreatitis, small diameter of the pancreatic duct (<3 mm) and massive blood loss (>500 mL) [14], [15], [16]. A high BMI is another risk factor probably through pancreatic steatosis which is often seen in obese Patients [14], [17], [18], [19]. Advanced age can also be associated with pancreatic steatosis [18]. Earlier, an increasing age was thought to be a risk factor for POPF [20], but this could not be verified in recent studies [21], [22]. Male gender is also mentioned by a number of authors as a risk factor [14], [22], [23]. Preoperative und postoperative risk factors which could be retrospectively evaluated were included as cofounders for our PSM.

In the literature there are many different anastomosis techniques for the pancreatojejunostomy after pancreatic head resection; Chromik [24] describes, that every center has an individual technique of the anastomosis which makes any comparison difficult. A number of techniques have been suggested to reduce the rate of POPF, which shows that there is no ideal technique. A number of reports on the modified Blumgart technique suggest superiority over the conventional pancreatojejunostomy and show it’s potential to reduce the rate of clinically relevant POPF and the adverse postoperative complications [3], [25].

In the first publication about the Blumgart anastomosis 187 patients who underwent a pancreatic head resection were examined. The authors reported 1.6% mortality and a very low POPF rate (grade B + grade C) of 6.9% [3]. Kleespies et al. compared the Blumgart technique with the modified Cattel-Warren anastomosis. After Blumgart anastomosis the study shows a significantly lower rate of POPF (4%), reduced duration of the operation, reduced postoperative hemorrhage, reduced length of stay on intermediate care units, and less surgical and general complications [25]. Li came to similar results with a lower rate of POPF and general complications by the Blumgart anastomosis [26]. A recent study shows the potential of the Blumgart anastomosis to reduce adverse complications and POPF “grade C” over the duct-to-mucosa anastomosis and likewise the adverse complications and the 90 day mortality over the invagination pancreatojejunostomy technique [27]. The first big multicenter randomized controlled study (RCT), which compares the conventional Cattell–Warren anastomosis with the Blumgart anastomosis should bring new awareness in the near future [28].

The analysis of the data until now shows, that the Blumgart anastomosis could reduce the rate of POPF. The superiority of the technique is particularly shown for soft pancreatic parenchyma, simple feasibility and reduced duration of the operation. Eventual future introduction of the minimally invasive technique into our hospital led us to introduce this technique in 2017. The aim of this study is to evaluate our experience with the Blumgart anastomosis in comparison with the conventional pancreatic anastomosis. With the help of PMS we created 2 groups (ratio 1:2) with balanced distribution of the known risk factors (see Table 1). We tried to produce two homogenous comparable study groups depending on the data which we had. Unfortunately, a number of risk factors with known influence on the rate of POPF for example the diameter of the pancreatic duct and texture of the parenchyma could not be retrospectively evaluated. Therefore, they could not be included in the PSM as cofounders. A selection bias can therefore not be excluded and this is the weakness of our study.

In the Blumgart-group we recorded a tendency to lower POPF (“biochemical Leak”, POPF “grade B” and POPF “grade C”) in comparison to the conventional anastomosis (PJ-group). This difference was not significant. In contrast the occurrence of the clinically adverse POPF grade C was significantly lower in the Blumgart-group (1 POPF grade C) in comparison to the PJ-group (12 POPF grade C). The duration of the operation was significantly lower in the Blumgart-group as in the PJ-group.

The shorter operation time has to be discussed carefully. On one hand the increasing experience of the surgeons is responsible for the shorter operation time. Further major changes in saving preparation time are based on the increasing use of ultrasound scalpels (Thunderbird^™^, Olympus) during the last years. On the other hand, in our opinion, also the faster performance of the anastomosis, especially in difficult situations (soft pancreas, small duct) due to the Blumgart technique is contributing essentially to reduce operation time.

A difference in the length of stay on intermediate care units could not be documented, also regarding the length of hospital stay, there was no difference between the two groups. Regarding the length of hospital stay there are a number of other influencing social factors, for example organization of admission dates in rehabilitation units for elderly patients or organization of home support. There is therefore no direct conclusions here.

With the help of propensity score matching we came up with two homogenous patient groups hence producing reliable results. The analysis hereafter shows that the Blumgart anastomosis is a safe pancreatic anastomosis because it has the potential to decrease the rate of adverse postoperative pancreatic fistula. Furthermore, a shorter length of the operation was recorded in the Blumgart-group. These results correspond to the already existing evidence.
