# Supplementary material for: The modified Blumgart anastomosis after pancreaticoduodenectomy: a retrospective single center cohort study
Source: Innov Surg Sci. 2020 Dec 21;5(3-4):105–9. doi: 10.1515/iss-2020-0021 (PMC7790181; doi:10.1515/iss-2020-0021)
Supplement: Supplementary file 1 [file iss-05-20200021-s001.pdf]

Reviewer Assessment

Georgi Kalev et. al: The modified Blumgart anastomosis after pancreaticoduodenectomy: a retrospective single center cohort study

Reviewers' Comments to Original Submission

Reviewer 1: anonymous

Date received: 21-Sep-2020
Reviewer recommendation: **Reject for the following reasons**
Reviewer overall scoring: **Low**

Assessment Form scores: 5 = High/Yes; 3 = Medium/Adequate; 1 = Low

Is the subject area appropriate for the journal				2	
Does the title clearly reflect the paper's content?			3		
Does the abstract clearly reflect the paper's content			3		
Do the keywords clearly reflect the paper's content?			3		
Does the introduction present the problem clearly?		4			
Are the results/ conclusions justified?					1
How comprehensive and up-to-date is the subject matter presented?				2	
How adequate is the data presentation?		4			
Are units and terminology used correctly?		4			
Is the number of cases adequate?					1
Are the experimental methods/ clinical studies adequate?			3		
Is the length appropriate in relation to the content?			3		
Does the reader get new insights from the article?				2	
Please rate the practical significance.					1
Please rate the accuracy of methods.			3		
Please rate the statistical evaluation and quality control.			3		
Please rate the appropriateness of the figures and tables.		4			
Please rate the appropriateness of the references.		4			
Please evaluate the writing style and use of language.		4			
Please judge the overall scientific quality of the manuscript.				2	
Are the methods used worthy of reproduction in greater deal?	No				
Would you be willing to review a revision of this manuscript?	No				

Comments to author: This manuscript is retrospectively comparing two techniques of pancreatic anastomosis (match-pair). The results demonstrate no difference apart from a reduction of grade c Fistula with the Blumgart-technique.

There are several concerns to be addressed: The Institution only performed 221 pancreatic head resections in 11 years (about 20 /year). Two different techniques have been used so that the expertise for each technique is low. Only 29 and 56 patients were included in the analysis. Thus, not enough patients were evaluated. The leakage rate (B and C Fistula) did not differ. The definition of leakage changed over the years and it has to be questioned whether the documentation allow to classify the correct type of fistula.
Several techniques have been compared in the past, and it is always important to know what the standard procedure was, how many surgeons actually performed the anastomosis, ...
The authors may explain why operations in which the Blumgart anastomosis was performed were quicker, since the Variation of a pancreatic head resection is not limited to the duration of the anastomosis.

Reviewer 2: anonymous

Date received: 14-Oct-2020
Reviewer recommendation: **Return to author for major modifications**
Reviewer overall scoring: **Medium**

Assessment Form scores: 5 = High/Yes; 3 = Medium/Adequate; 1 = Low

Is the subject area appropriate for the journal			3		
Does the title clearly reflect the paper's content?		4			
Does the abstract clearly reflect the paper's content		4			
Do the keywords clearly reflect the paper's content?		4			
Does the introduction present the problem clearly?		4			
Are the results/ conclusions justified?			3		
How comprehensive and up-to-date is the subject matter presented?			3		
How adequate is the data presentation?			3		
Are units and terminology used correctly?		4			
Is the number of cases adequate?			3		
Are the experimental methods/ clinical studies adequate?			3		
Is the length appropriate in relation to the content?			3		
Does the reader get new insights from the article?				2	
Please rate the practical significance.			3		
Please rate the accuracy of methods.		4			
Please rate the statistical evaluation and quality control.		4			
Please rate the appropriateness of the figures and tables.			3		
Please rate the appropriateness of the references.			3		
Please evaluate the writing style and use of language.			3		
Please judge the overall scientific quality of the manuscript.			3		
Are the methods used worthy of reproduction in greater deal?	Yes/No				
Would you be willing to review a revision of this manuscript?	Yes/No				

Comments to author:
This manuscript is well written and the analysis accurate. I am happy to suggest some additional information that may increase the value of a potential publication: 1. In the abstract/discussion delete the word promising because this technique

is not new. Also the last sentence regarding PRT as it used standard in many manuscripts and the chances for such a study are very low

2. Interesting would be a suggestion regarding which patients may benefit from this type of anastomosis (high risk pancreas: soft and small duct??)

3. Even if there is no solid own data on this please to try to find out, if the pancreas was high or low risk for anastomosis, for instance by looking for information in the OR notes (duct enlarged/small, pancreas high or low volume, consistency) and create a possible correlation to your findings

4. Add in discussion the possible impact of the learning curve with this anastomosis (how many surgeons, tutorial, mentoring?)

5. In the table 2 please calculate the percentanges as well. In this case a mortality of 10% has to be discussed: why did the patients die in every group, how was the „failure to rescue"

6. Please indicate if this data led to a change of the anastomis standard in the hospital

Authors' Response to Reviewer Comments

Date received: 03-Nov-2020

Response to reviewer 1

1. „The Institution only performed 221 pancreatic head resections in 11 years (about 20 /year). Two different techniques have been used so that the expertise for each technique is low. Only 29 and 56 patients were included in the analysis. Thus, not enough patients were evaluated".

We are a certified pancreatic center of the DKG (Deutsche Krebsgesellschaft, German Cancer Society) and performed the required minimum quantity of 20 pancreatic resections each year. As we are the referral hospital in a holding (RKH regional hospital holding) of 9 hospitals in the south of Germany, all pancreatic resections of this area are referred to our pancreas center, and we performed on average 34 pancreatic resections (24 resections for cancer) per year in the last years. Thus we are continuously in the focus of a rigid quality measurement program, which we fulfil every year with less than 10% revision operations after pancreatic resections and a mortality rate below 5%. Only 2 pancreatic surgeons are certified and present during every pancreatic resection. Further the minimum volume where risk of death would fall below the average mortality of 8.8% was calculated as 29 cases per year (Nimptsch U, Mansky T: Hospital volume and mortality for 25 types of inpatient treatment in German hospitals: observational study using complete national data from 2009 to 2014. BMJ Open 2017;7:e016184. doi: 10.1136/bmjopen-2017-016184)

We included this fact in „Materials and methods".

2. „The leakage rate (B and C Fistula) did not differ. The definition of leakage changed over the years and it has to be questioned whether the documentation allow to classify the correct type of fistula.".

The leakage rate of low grade fistula indeed did not change. Grade C fistula rate was significantly different between the two groups, which clinically is mainly responsible for severe complications, like bleeding by arrosion. The postoperative fistula were classified according to the latest definitions during our retrospective data research. We have studied two historical cohorts.

We cleared that out in „Materials and methods

3. „Several techniques have been compared in the past, and it is always important to know what the standard procedure was, how many surgeons actually performed the anastomosis"

Until September 2017, conventional end-to-side pancreatojejunostomy was our standard anastomosis. From September 2017 until today, the modified Blumgart anastomosis represents anastomosis standard in our hospital.

We added this fact in „material and methods“

4. „The authors may explain why operations in which the Blumgart anastomosis was performed were quicker, since the Variation of a pancreatic head resection is not limited to the duration of the anastomosis.“

Indeed the shorter operation time has to be discussed carefully. On the one hand the increasing experience of the surgeons is responsible for the shorter operation time. Further major changes in saving preparation time came by increasing use of ultrasound scalpels (Olympus Thunderbird) parallel during the last years. On the other hand, in our opinion, this is due to the faster performance of the anastomosis, especially in difficult situations (soft pancreas, small duct) due to the Blumgart technique.

We added the facts in „discussion“

Response to reviewer 2:

1. „In the abstract/discussion delete the word promising because this technique is not new. Also the last sentence regarding PRT as it used standard in many manuscripts and the chances for such a study are very low“

We deleted the mentioned sentences

2. „Interesting would be a suggestion regarding which patients may benefit from this type of anastomosis (high risk pancreas: soft and small duct?)“

Our literature research showed that patient with soft pancreas and small duct might benefit from the Blumgart anastomosis technique. This fact was already included in the discussion.

3. „Even if there is no solid own data on this please to try to find out, if the pancreas was high or low risk for anastomosis, for instance by looking for information in the OR notes (duct enlarged/small, pancreas high or low volume, consistency) and create a possible correlation to your findings“

As we checked every single OR report carefully, there was no correlation.

4. „Add in discussion the possible impact of the learning curve with this anastomosis (how many surgeons, tutorial, mentoring?)

As mentioned above in point 1 of reviewer 1, we added the details about surgeons and mentoring in the “Materials and methods”.

5. „In the table 2 please calculate the percentages as well. In this case a mortality of 10% has to be discussed: why did the patients die in every group, how was the „failure to rescue ‘“

We didn’t analyze representative samples but subgroups with certain demographic and clinical characteristics. Therefore, the calculation of a percentage mortality is not allowed in this context, as it leads to false conclusions about the total mortality rate.
The details were added in the legend of the table 2.
As explained in point 1 for reviewer 1, we are under quality control and perform well with an overall mortality rate below 5%. We have 24/7 full interventional endoscopy (including transgastric drainage etc.) and angiographic interventions (including coiling and stenting of hepatic arteries). Regarding the mortality rate, we reviewed every chart thoroughly, especially in terms „failure to rescue“ and we found no evidence for “failure to rescue”.

6. „Please indicate if this data led to a change of the anastomosis standard in the hospital"

As our mortality rate was constant over the years, this data did not lead to the change of the anastomotic technique. As we thought about laparoscopic or robotic pancreatic resection some years ago, we were looking for a simple and safe anastomosis, which we found in the Blumgart anastomosis. Due to the discussion in the literature, we did not start laparoscopic or robotic right sided pancreatic resections, but are gaining good results with our robotic assisted left sided pancreatic resections.

Reviewers' Comments to Revised Submission

Reviewer 2: anonymous

Date received: 17-Nov-2020
Reviewer recommendation: **Accept in present form**
Reviewer overall scoring: **Medium**

Assessment Form scores: 5 = High/Yes; 3 = Medium/Adequate; 1 = Low

Is the subject area appropriate for the journal			3		
Does the title clearly reflect the paper's content?		4			
Does the abstract clearly reflect the paper's content		4			
Do the keywords clearly reflect the paper's content?		4			
Does the introduction present the problem clearly?		4			
Are the results/ conclusions justified?			3		
How comprehensive and up-to-date is the subject matter presented?			3		
How adequate is the data presentation?			3		
Are units and terminology used correctly?		4			
Is the number of cases adequate?			3		
Are the experimental methods/ clinical studies adequate?			3		
Is the length appropriate in relation to the content?			3		
Does the reader get new insights from the article?				2	
Please rate the practical significance.			3		
Please rate the accuracy of methods.		4			
Please rate the statistical evaluation and quality control.		4			
Please rate the appropriateness of the figures and tables.			3		
Please rate the appropriateness of the references.			3		
Please evaluate the writing style and use of language.			3		
Please judge the overall scientific quality of the manuscript.			3		
Are the methods used worthy of reproduction in greater deal?	No				
Would you be willing to review a revision of this manuscript?	Yes				

Comments to author:

Thank you for considering our recommendations

Reviewer 3: anonymous

Date received: 20-Nov-2020
Reviewer recommendation: **Accept in present form**
Reviewer overall scoring: **High**

Assessment Form scores: 5 = High/Yes; 3 = Medium/Adequate; 1 = Low

Is the subject area appropriate for the journal	5				
Does the title clearly reflect the paper's content?		4			
Does the abstract clearly reflect the paper's content		4			
Do the keywords clearly reflect the paper's content?		4			
Does the introduction present the problem clearly?		4			
Are the results/ conclusions justified?			3		
How comprehensive and up-to-date is the subject matter presented?		4			
How adequate is the data presentation?			3		
Are units and terminology used correctly?		4			
Is the number of cases adequate?			3		
Are the experimental methods/ clinical studies adequate?		4			
Is the length appropriate in relation to the content?		4			
Does the reader get new insights from the article?			3		
Please rate the practical significance.	5				
Please rate the accuracy of methods.		4			
Please rate the statistical evaluation and quality control.		4			
Please rate the appropriateness of the figures and tables.		4			
Please rate the appropriateness of the references.					
Please evaluate the writing style and use of language.		4			
Please judge the overall scientific quality of the manuscript.		4			
Are the methods used worthy of reproduction in greater deal?					
Would you be willing to review a revision of this manuscript?	No				

Comments to author:

Being the reviewer after the 1st revison of the manuscript I feel that the paper substantially improved. Still, there is some concern about he number of cases in particular in the Blumgart-group. However, according to the own experiences, the Blumgart technique is a nice option to reduce postoperative pancreatic fistulas. this may increase patients' safety, so that I recommed the manuscript for publication in the present form.
